# SGLT 2 Inhibitors; glycemic control, weight loss and safety profile in patients with type 2 Diabetes, at Medicell Institute (MIDEM)

**DOI:** 10.12669/pjms.37.1.2701

**Published:** 2021

**Authors:** Erum Sohail, Tasnim Ahsan, Saima Ghaus, Wasfa Aijaz

**Affiliations:** 1 Dr. Erum Sohail, FCPS, MBBS. Medicell Institute of Diabetes Endocrinology & Metabolism (MIDEM), 9E, 8^th^Zamzama Commercial Lane, Phase-V, DHA, Karachi, Pakistan; 2 Prof. Tasnim Ahsan, MRCP(UK), FRCP(Glasg), FRCP(Edin), FRCP(Lon). Medicell Institute of Diabetes Endocrinology & Metabolism (MIDEM), 9E, 8^th^Zamzama Commercial Lane, Phase-V, DHA, Karachi, Pakistan; 3 Dr. Saima Ghaus, FCPS, MBBS. Medicell Institute of Diabetes Endocrinology & Metabolism (MIDEM), 9E, 8^th^Zamzama Commercial Lane, Phase-V, DHA, Karachi, Pakistan; 4 Dr. Wasfa Aijaz, FCPS, MBBS. Medicell Institute of Diabetes Endocrinology & Metabolism (MIDEM), 9E, 8^th^Zamzama Commercial Lane, Phase-V, DHA, Karachi, Pakistan

**Keywords:** SGLT 2 Inhibitors, Efficacy, Safety, Glycemic control

## Abstract

**Background & Objective:**

Sodium glucose co-transporter-2 inhibitors (SGLT 2 inhibitors) are newer anti-hyperglycemic agents, which improve glycemic control independent of insulin secretion with a low risk of hypoglycemia. This study aimed to assess the efficacy of SGLT 2 inhibitors in terms of glycemic control, weight reduction and safety profile in our patients with type 2 Diabetes (T2D).

**Methods:**

This is a prospective analysis, conducted at Medicell Institute of Diabetes, Endocrinology and Metabolism (MIDEM), Karachi Pakistan from January 2018 till July 2019. This study included patients with T2D, who were treated with SGLT 2 inhibitors add on to other anti-diabetic drugs. Baseline and follow up weight, BMI, HbA1c, blood pressure (BP), renal function and side effect profile was assessed.

**Results:**

Study included 140 patients; 53% females and 47% males. Mean Age was 55.6 ± 10.3 years. Mean weight at baseline was 81.5 ±16.5 kg. Mean duration of T2D was 10.3 ± 6.75 years, with a mean HbA1C at baseline of 9.1± 1.6%. Follow up data was available for 90 patients at the time of analysis. HbA1C improved considerably to 7.6± 0.9 (P< 0.001) and mean weight reduced to 78.5 ± 16.1 kg (P≤0.003), at first follow-up.

**Conclusion:**

Dapagliflozin and Empagliflozin offer a significant additional drug in improving glycemic control with the additional advantage of weight loss and hypoglycemia safety.

## INTRODUCTION

T2D is a major global public health concern. Just fewer than half a billion people are living with diabetes worldwide and the number is projected to increase by 25% in 2030 and 51% in 2045.^1^Apart from inter-ethnic differences in genetic predisposition, Asian patients with T2D have several distinctive features; high prevalence of young-onset diabetes,^2^ metabolic syndrome, β-cell dysfunction,^3^ and a higher degree of insulin resistance (particularly in South Asians).^4^,^5^ They also have lower lean muscle mass, higher visceral fat mass, lower circulating adiponectin levels,^6^ and are more likely to exhibit postprandial hyperglycaemia.^7^According to IDF 2019 estimates, prevalence of diabetes in Pakistan is reported as 19.4 million,^1^ whereas two recent national surveys reported prevalence of diabetes as 26.4% and 16.9%, and pre-diabetes prevalence of 14.4% and 10.9%, respectively.^8^,^9^ There is a continuing unmet need for novel glucose lowering therapies that provide durable glycemic control while avoiding hypoglycemia, weight gain, fluid retention and prevention of diabetes related cardiovascular and renal complications, which are recognized problems with a number of existing glucose lowering drugs.^10^-^12^

SGLT 2 inhibitors are newer anti-hyperglycemic agents, which improve glycemic control independent of insulin secretion with a low risk of hypoglycemia.^13^ There is limited data on use of SGLT 2 inhibitors in Pakistan; this is a case series of local patients treated with SGLT 2 inhibitors. This study aimed to assess the efficacy of SGLT 2 inhibitors in terms of glycemic control, weight reduction and safety profile in cohort of Pakistani patients with T2D.

## METHODS

This is a prospective analysis conducted at Medicell Institute of Diabetes, Endocrinology and Metabolism (MIDEM), Karachi Pakistan from January 2018 till July 2019. This study was approved by the ethical committee of MIDEM (IRB – 004/MHS/19).

Patients with T2D, who need escalation of treatment for failing to achieve HbA1c target on usual anti-diabetic drugs or a change of anti-diabetic medication required for purpose of cardiovascular and renal protective benefits of this class of drugs. All patients were counseled about the side effects and the need to report immediately if there were symptoms related to urinary, vulvo-vaginal or perineal infections.

### Inclusion Criteria:

1. Patients with T2D, who needed escalation of treatment for failing to achieve HbA1c target on standard anti-diabetic medication, including insulin (HbA1c > 6.5).
2. Drug naive patients who meet ADA/EASD guideline specified criteria for using SGLT 2inhibitors, i-e cardiovascular disease (CVD), chronic kidney disease (CKD) and Obesity
3. Patients on other anti-diabetic medications with existing CVD or mild CKD (eGFR < 90 and > 30 mL/min/1.73m2) for beneficial effects of SGLT 2inhibitors.

### Exclusion Criteria

Patients with CKD, if eGFR < 30 mL/min/1.73m.^2^ Patients with history of genital mycotic infections, recurrent urinary tract infections (UTIs), pyelonephritis, acute illness, T1D, pregnant or lactating women and history of diabetic ketoacidosis (DKA) in last 6 months.

Baseline weight, BMI, HbA1c, fasting and random blood sugar, blood pressure, renal function and duration of diabetes were assessed.

The preexisting anti diabetic medication included Metformin, Sulphonylurea, DPP4 Inhibitors, Thiazolidinediones, GLP 1 Analogues or Insulin. The patients on insulin were either continued with the pre-existing dose of insulin or dose was reduced if there was risk of hypoglycemia. Likewise, dose of sulphonylurea was also reduced and was stopped in a few patients with hypoglycemia risk. Patients were advised to check blood glucose at-least twice per week and if they were unwell or had symptoms suggestive of hypoglycemia occurs. Hypoglycemia is defined as a blood sugar level below 70 mg/dl with or without symptoms. Diet and lifestyle were intensified.

The commercially available SGLT 2 inhibitors in Pakistan at the time of this study were Dapagliflozin 5- 10 mg and Empagliflozin 10-25 mg, and either of them were prescribed once daily in the morning, in addition to other anti-diabetic drugs. The duration of follow up was 3-6 months. Changes in weight, HbA1c, FBS, blood pressure, Cholesterol levels and Creatinine along with side effect profile were analyzed on follow up.

### Statistical analysis

Frequency and percentages were presented for categorical variables that include gender, treatment assigned and adverse reactions reported during follow up visits. Mean and standard deviation was illustrated for age, weight, BP, T2D Duration, HbA1C and other numerical variables. Mean difference of HbA1c, SBP, DBP, weight, FBS from baseline to follow-up (3-6 months) was analyzed by applying paired sample T-test through SPSS v.21, considering p-value <0.001 as statistically highly significant.

## RESULTS

Our Study included a total of 140 patients; 53% were females and 47% were males. Baseline characteristics are given in Table-I. Mean Age was 55.6 ± 10.3 years. Mean weight at the time of starting SGLT 2i was 81.5 ±16.5 kg; mean BMI was 31.9 ± 7.7 kg/m^2^. Mean systolic blood pressure at baseline was 144.1± 19.5 mmHg and diastolic blood pressure was 83.9± 10.5 mmHg. Mean duration of T2D was 10.3 ± 6.75 years, with a mean HbA1C at baseline of 9.1± 1.6%. Baseline creatinine of our study group was 0.9 mg/dl and eGFR was 127.92 mL/min/1.73m^2^. In our study group, 58 (41.4%) patients were overweight/obese, 18 (12.8%) had Ischemic Heart Disease (IHD) and 27 (19.2%) patients had CKD. The patients were on different anti-diabetic medications: 87.1% on Metformin, 75.7% on DPP-4 inhibitors, 31.4% on Sulphonylureas, 26.4% on Insulin, 7.9% on Pioglitazone and 6.5% were on GLP-1 agonist. Patients received two types of SGLT 2 inhibitors; 54.3% were given Dapagliflozin in the dose of 5-10mg per day, while 45.7% were given Empagliflozin in the dose of 10-25mg per day.

**Table-I:** Baseline characteristics of study participants (n=140).

***Gender***	
Female	75 (53%)	
Male	65 (47%)	
Age(years), n= 124	55.6	±10.3
Female (n=66)	57.03	±10.4
Male (n=55)	53.9	±10.1
Weight (Kg)	81.5	±16.5
Body Mass Index (Kg/m^2^)	31.9	±7.7
Duration of Diabetes Mellitus (years)	10.3	±6.75
Systolic Blood Pressure (mmHg)	144.1	±19.5
Diastolic Blood Pressure (mmHg)	83.9	±10.5
Heart Rate (Bpm)	88.0	±10.9
HbA1c (%)	9.1	±1.6
Fasting Blood Sugar (mg/dL)	190.3	±57.3
Random Blood Sugar (mg/dL)	255.7	±59.8
Cholesterol (mg/dL)	169.5	±42.4
Triglyceride (mg/dL)	220.3	±161.7
High-Density Lipoproteins (mg/dL)	38.2	±7.8
Low-Density Lipoproteins (mg/dL)	99.7	±35.7
Hemoglobin (mg/dL)	12.9	±1.58
Alanine Aminotransferase (IU/L)	30.5	±18.2
Creatinine (mg/dL)	0.93	±0.36

**Graph.1:**
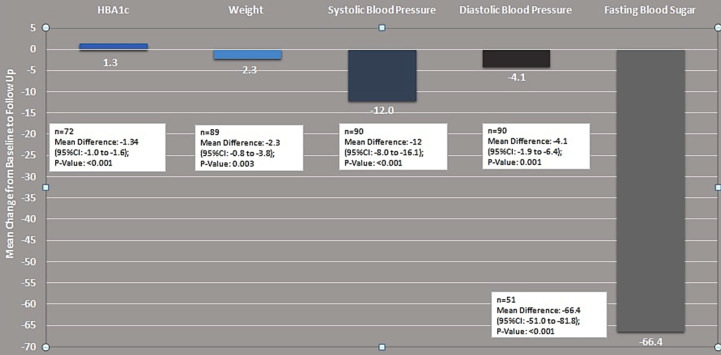
Effect of SGLT2 Inhibitors. (Empagliflozin & Dapagliflozin) on Systolic Blood Pressure, Diastolic Blood Pressure, Weight, Fasting, Blood Sugar, Random Sugar from to Follow Up.

**Graph.2:**
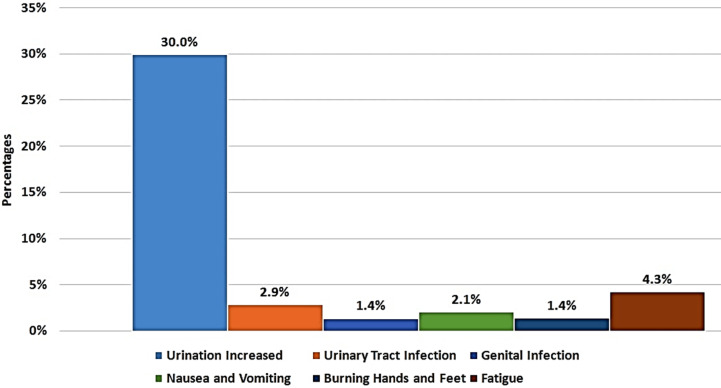
Patients Reported Adverse Events with SGLT2 Inhibitors (Empagliflozin &Dapagliflozin) During Follow Up Visit (n=140).

Follow up data was available for 90 patients at the time of analysis. Fifty Patients were lost to follow, approximately 40 % of these patients, were still taking the drug but could not come and get the test done due to financial constraints or other issues. HbA1C improved considerably to 7.6± 0.9 (P≤ 0.001), at first follow-up between three to six months. Mean weight reduced to 78.5 ±16.1 (P≤ 0.003). There was significant reduction in systolic and diastolic blood pressures (Table-II) (Graph.1); with no deterioration of renal function (creatinine was 0.87 mg/dl). The drug was discontinued in three patients; one had genital infection and 2 had excessive fatigue. Forty-two patients (30%) reported increased frequency of urine; six patients (4.3%) had fatigue, four patients (2.9%) had UTI, followed by nausea and vomiting (2.1%) and genital infection and burning hands and feet were reported in 1.4% (Graph.2). No patient reported symptoms or documented hypoglycemia or euglycemic DKA.

**Table-II:** Effect of SGLT2 Inhibitors on HbA1c, Systolic Blood Pressure, Diastolic Blood Pressure, Weight, Fasting Blood Sugar from Baseline to Follow Up.

	Baseline (Mean ±SD)	Follow up (Mean ±SD)	Mean Difference	P-value

HbA1c (n=72)	8.9 ±1.6	7.6± 0.9	1.3 ±1.3	<0.001
SBP (n=90)	146.8± 19.8	134.8± 16.4	12.1 ±19.4	<0.001
DBP (n=90)	85 ±11.0	80.8 ±8.8	4.1 ±10.6	<0.001
Weight (n=89)	80.8 ±18.1	78.5 ±16.1	2.3 ±6.9	0.002
Fasting Blood Sugar (n=51)	202.2 ±59.4	135.7 ±23.5	66.4 ±54.7	<0.001

Paired t-test was applied for statistical significance.

## DISCUSSION

With the increasing prevalence of T2D and associated risk of CVD and CKD, SGLT 2 inhibitors represent a key therapeutic addition for clinicians in the management of patients with diabetes. Our study demonstrates that when SGLT 2 inhibitors were added at any stage of the disease in the real world scenario, in patients with uncontrolled T2D, there is a clinical and statistically significant improvement in glycemic control, weight loss and reduction in blood pressure in our patients, as has been demonstrated in the landmark studies with SGLT 2 inhibitors.^14^,^15^ This class of drug improves glycemic function independent of insulin secretion. In addition SGLT 2 inhibitors exert favorable effects on multiple risk factors including blood pressure, body weight and renal function; in addition they also provide an opportunity to reduce the risk of cardiovascular disease (CVD) in patients with T2D.^14^,^15^ Results of the EMPA-REG OUTCOME TRIAL, demonstrated a relative risk reduction of 38%, 35% and 32% with respect to death from a cardiovascular event, hospitalization due to heart failure (HHF) and all-cause mortality respectively.^16^ Cardiovascular outcome trails (CVOTs) with other SGLT 2 inhibitors have also shown similar effects on cardiovascular complications along with a delay in renal function deterioration indicating an overall beneficial class effect.^15^,^17^,^18^A recent study has demonstrated beneficial effects of Dapagliflozin in heart failure in patients without diabetes, mandating a significant change in the guidelines for heart failure management.^19^

There is a paucity of data from South Asian countries specially Pakistan. This study aims to evaluate the effects and tolerability of this class of drugs in our population. In an Indian study, the mean HbA1c reduction and weight reduction observed was 1.02±0.24% and 2.64±1.27 kg respectively.^20^ In another study from North India, HbA1c reduction observed was 1.8 %, while weight reduced by −3.45 kg.^21^ According to a recent meta-analysis of treatment with SGLT 2 inhibitors in Asian and non-Asian patients, reduction in HbA1c levels was more in Asians than in non-Asians, with the difference of 0.05% between groups but was not significant (P> 0.05).^22^ In a small study of Dapagliflozin in Turkish patients, HbA1c improved 1.6% with significant weight reduction and decrease in insulin dose.^23^ A recent publication on use of Dapagliflozin in Pakistan, reported mean HbA1c and weight reduction of -1.1% and -1.98 kg, respectively.^24^ Our study results are comparable to above studies with mean HbA1c reduction of 1.34% and mean weight reduction of 2.3 kg.

SGLT 2i have additional advantage of blood pressure reduction. This effect is multifactorial mainly attributable to volume contraction by natriuretic and diuretic effects, weight loss, and better arterial compliance (elasticity index of large arteries).^25^ A meta-analysis which included 27 randomized controlled trials (n, 12 960) showed that SGLT 2 inhibitors significantly reduced both systolic (weighted mean difference, −4.0 mm Hg)and diastolic blood pressure (weighted mean difference, −1.6 mm Hg; [95% CI, −1.9-−1.3].^26^ Patients in our study achieved similar magnitude of reduction in systolic and diastolic blood pressures.

SGLT 2 inhibitors are often associated with a risk of genital mycotic and lower UTI due to increased urinary excretion of glucose; these side effects being observed more often in women than in men.^27^ High risk of genital mycotic infections has been reported in a cohort of Indian patients with T2D on SGLT 2 inhibitors, one episode of genital mycotic infection occurred in 25.9% of patients and 12.2% had a second episode.^28^ The incidence of UTI and vulvo-vaginal candidiasis was very low in our study. Only two patients had genital infection and in one led to drug withdrawal, no case of perineal fasciitis was seen in this cohort. No patient reported symptoms of hypoglycemia or euglycemic DKA. This may be attributed to the fact, that we counseled our patients to increase water intake and the importance of maintaining good personal hygiene to avoid mycotic infections. Another Indian study, has reported similarly low incidence of adverse effects (AEs), including genitourinary AEs.^26^

Clinical studies have demonstrated that SGLT 2 inhibitors often induce an initial decrease in eGFR in patients with T2D; subsequently eGFR remains stable over time and returns toward baseline during continuous SGLT 2 inhibition.^14^,^15^ This initial increase in eGFR was noted in 23 (25%) patients in our cohort.

### Limitations of the study

Limitation of our study is its small sample size and inability to report on the follow up of all patients prescribed SGLT 2 inhibitors in this cohort. We did not compare the effect of SGLT 2 inhibitors with other anti-diabetic drugs in a controlled manner. Since this was a short study, we did not assess the effect of SGLT 2 inhibitors on long term cardiovascular and renal outcomes.

## CONCLUSION

Mean reduction of HbA1c (-1.34%) showed that Dapagliflozin and Empagliflozin offer a significant additional drug for improving glycemic control with the additional advantage of hypoglycemia safety and cardiovascular and renal benefit without any significant side effects in our population.

### Author`s Contribution:

**ES:** Contributed to conception and design of the study, Data acquisition, analysis and interpretation, Drafting the article and Final approval of the version to be published and responsible and accountable for this study.

**TA:** Accuracy and Integrity of the work, Contribution to conception and design of the study, Patient management, Critical analysis of the article and Final approval of the version to be published

**SG:** Contributed to conception and design of the study, Data acquisition and collection and Revision of article.

**WA:** Conception and design of study and Data acquisition and collection.
